# Collagen Hydrogels Loaded with Silver Nanoparticles and Cannabis Sativa Oil

**DOI:** 10.3390/antibiotics10111420

**Published:** 2021-11-20

**Authors:** Pablo Edmundo Antezana, Sofia Municoy, Claudio Javier Pérez, Martin Federico Desimone

**Affiliations:** 1 Universidad de Buenos Aires, Consejo Nacional de Investigaciones Científicas y Técnicas (CONICET), Instituto de Química y Metabolismo del Fármaco (IQUIMEFA), Facultad de Farmacia y Bioquímica, Junín 956, Buenos Aires 1113, Argentina; pablo.e.antezana@gmail.com (P.E.A.); smunicoy@gmail.com (S.M.); 2 Grupo Ciencia y Tecnología de Polímeros, Instituto de Investigaciones en Ciencia y Tecnología de Materiales (INTEMA), Universidad Nacional de Mar del Plata, Juan B. Justo 4302, Mar del Plata 7600, Argentina; cjperez@fi.mdp.edu.ar

**Keywords:** collagen, biomaterials, silver nanoparticles, *Cannabis sativa*, wound healing, antimicrobial

## Abstract

Wounds represent a major healthcare problem especially in hospital-associated infections where multi-drug resistant strains are often involved. Nowadays, biomaterials with therapeutic molecules play an active role in wound healing and infection prevention. In this work, the development of collagen hydrogels loaded with silver nanoparticles and *Cannabis sativa* oil extract is described. The presence of the silver nanoparticles gives interesting feature to the biomaterial such as improved mechanical properties or resistance to collagenase degradation but most important is the long-lasting antimicrobial effect. *Cannabis sativa* oil, which is known for its anti-inflammatory and analgesic effects, possesses antioxidant activity and successfully improved the biocompatibility and also enhances the antimicrobial activity of the nanocomposite. Altogether, these results suggest that this novel nanocomposite biomaterial is a promising alternative to common treatments of wound infections and wound healing.

## 1. Introduction

Loss of skin integrity because of injury or illness may result in substantial physiologic imbalance. In addition, different factors such as repeated trauma or underlying diseases can affect the normal wound-healing cascade. In some cases, the loss of the skin barrier function due to impaired wound healing facilitates the development of microbial communities leading to infection of dermal wounds. These open wounds represent a major healthcare problem especially in hospital-associated infections where multi-drug resistant strains are often involved.

A broad range of biomaterial scaffolds has been developed to facilitate the restoration of the skin tissue favoring cellular attachment, proliferation, and differentiation [1,2,3]. The goal is to restore the functional and structural properties of the wounded tissue to the before injury levels.

Regardless of the specific biomaterial product, research orientation is towards medicated dressings for drug delivery. In this sense, the incorporated therapeutic molecules must play an active role in wound healing and infection prevention. Therefore, during the last few years, different alternatives have been proposed to this end [4]. These include different biomaterials (i.e., collagen, chitosan) in combination with antibiotics, nanoparticles, antimicrobial peptides, or natural products [5,6,7,8,9].

Collagen-based biomaterials are broadly used to treat wounds as collagen favors wound healing, is biocompatible and biodegradable [10]. Collagen hydrogels possess a highly porous structure and fibrillar network that favors cell migration and colonization. However, collagen hydrogels are mechanically fragile and have poor drug delivery systems since the drug content is usually released within minutes because of the wide porosity. Hence, the combination with inorganic nano-drug delivery systems can contribute to overcoming both disadvantages. On one hand, nanoparticles can work as inorganic fillers and contribute to improving the mechanical properties of hydrogels. On the other hand, nano delivery systems contribute to controlling and delaying the release of therapeutic agents.

Among the therapeutic agents, metal nanoparticles have been widely employed in biomaterials design mainly due to their antimicrobial activity [11,12,13]. In this sense, silver nanoparticles (AgNPs) have a well-known deleterious effect over a broad spectrum of Gram-positive and Gram-negative bacteria, including antibiotic-resistant ones [14]. Indeed, AgNPs are effective against *Staphylococcus aureus* and *Pseudomonas aeruginosa* which are the most frequent bacteria responsible for wound infection and biofilm formation [7]. The mechanism of silver antimicrobial activity involves the action over multiple targets including protein binding, nucleic acids complexation, inhibition of cell division, and reproduction. Altogether, these effects contribute to hindering the emergence of resistant strains.

Alternatively, natural products like the extracts obtained from plants emerged as true candidates in wound healing local therapies, mainly due to their antioxidant, antimicrobial and anti-inflammatory properties [15]. An interesting feature of plant extracts is that their therapeutic effect would be achieved by the synergistic action of various phytochemical compounds.

*Cannabis sativa* L. (hemp) is a plant of the *Cannabaceae* family. The flowered tops contain cannabinoids which have various molecular targets, such as cannabinoid receptors and other components of the endocannabinoid system. Recent works demonstrated the efficacy of cannabis-based therapies for treating wound-related pain, inflammation, and wound healing [16,17]. Wang et al., reported that the activation of cannabinoid receptors reduces inflammation, fibrogenesis, and stimulates reepithelialization during skin wound healing [18]. Furthermore, there has also been great interest in the antibacterial activity of cannabinoids since 1950 and plenty of works have demonstrated their bacteriostatic and bactericidal effect predominantly against Gram-positive bacteria [19,20,21,22].

Actually, the search for innovative medicated dressings is focused on the development of multiple drug delivery systems which provide suitable drug concentration in the wound, the reduction of dosing intervals, and the prevention of drug side effects. Indeed, the field of biomaterials combined with antimicrobials, antioxidants, and anti-inflammatory drugs has experienced a growing development. Particularly, the incorporation of silver nanoparticles in different types of dressings to induce wound healing has resulted in many published works [11]. For example, Mogrovejo-Valdivia et al., designed a wound dressing loaded with silver and ibuprofen. The biomaterial displayed a sustained release of ibuprofen and antibacterial activity against *E. coli* and *S. aureus* [23]. As well as this, a new dermal device of polyvinyl alcohol (PVA) containing AgNPs associated with a collagen–hyaluronic acid membrane has been recently developed to promote skin regeneration, control inflammation, and reduce fungal and bacterial proliferation [24]. Besides, Haidari et al. also reported a pH-responsive hydrogel for the controlled release of AgNPs in infected wounds [25].

In this work, the development of collagen hydrogels loaded with silver nanoparticles and *Cannabis sativa* oil extract is described. After the addition of an aqueous suspension of silver nanoparticles, collagen scaffolds containing 67 ± 7 mg/g of AgNPs were obtained. This new hybrid biomaterial exhibited prolonged antimicrobial activity against Gram-positive and Gram-negative bacteria. In fact, the new scaffold exhibited a bactericidal effect for seven days. Furthermore, the hydrogel loaded with AgNPs presented enhanced mechanical properties and greater stability against enzymatic collagenase degradation. In order to improve the biocompatibility of the antimicrobial collagen gel, *Cannabis sativa* was then added, and cytotoxicity against eukaryotic cells was significantly decreased. Then, this novel nanocomposite biomaterial is a promising alternative to common treatments of wound infections and wound healing: it avoids the proliferation of harmful bacteria in the affected zone, it provides a matrix with suitable mechanical properties to allow the growth of new tissue, and it is also highly biocompatible.

## 2. Results and Discussion

### 2.1. Synthesis and Characterization of Silver Nanoparticles

AgNPs were prepared by the borohydride reduction method as previously described [6]. For this, a capping agent was used to prevent agglomeration and stabilize the nanoparticles in the aqueous suspension. Although natural polymers have been demonstrated to have plenty of advantages for pharmacological and bionanomedical applications [26,27], plant-based compounds involve time-consuming extraction processes and may have microbial contamination. In contrast, synthetic polymers have great advantages over natural polymers, since they can be adapted to offer a wide range of possibilities with different kinds of products [28]. In this sense, we used PVA as the capping agent for AgNPs stabilization. PVA is one of the most used synthetic polymers as a stabilizer agent due to its good biocompatibility, no carcinogenicity, hydrophilicity, non-toxicity, and biodegradability in human tissues and fluids [29]. Thus, in the biomedical field, it is widely used to prevent nucleation and slow crystal growth [30].

**Figure 1:**
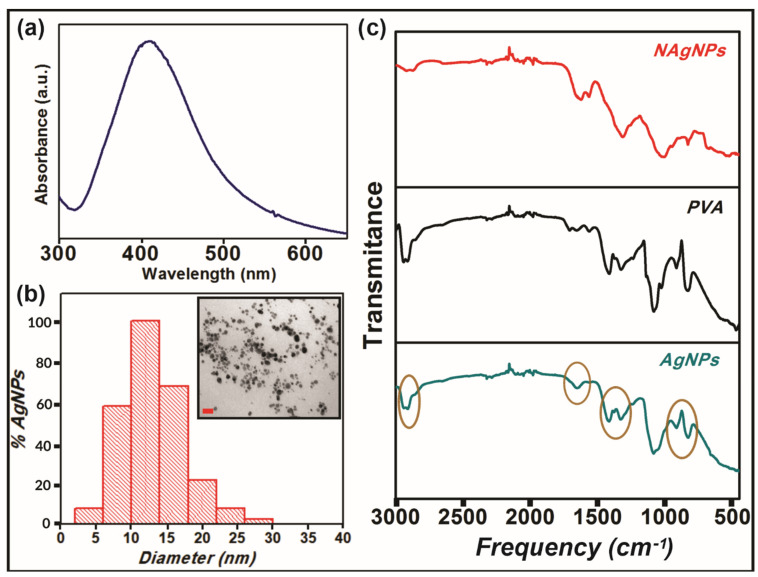
Characterization of silver nanoparticles (AgNPs). (**a**) The plasmon of AgNPs was measured by UV–vis spectroscopy. (**b**) Size distribution and TEM image (inset) of AgNPs. Scale bar represents 50 nm. (**c**) FTIR spectra of nude (NAgNPs, red) and modified (AgNPs, blue) silver nanoparticles and PVA (black).

Once the reaction was completed and a dark brown suspension was obtained, to confirm the formation of AgNPs, a UV-visible spectrum was performed, as silver nanoparticles are known to exhibit a characteristic surface plasmon resonance band in the range of 400–450 nm [31]. Figure 1a shows the distinctive surface plasmon of AgNPs with a maximum around 400 nm that also corroborates the nanometric dimensions of the particles [32]. Furthermore, the gaussian shape and intensity of the peak evidence the spherical geometry of the particles [33]. This was confirmed by Transmission Electron Microscopy (TEM), although Dynamic Light Scattering (DLS) is another widely used technique to study polydispersity and size distribution of nanoparticles. Transmission electronic microscopy is the preferred technique to take direct images and obtain information about the size, shape, and size distribution of nanoparticles [34,35]. On the other hand, DLS measures the hydrodynamic diameter of nanoparticles in a solution that is usually higher than the particle size. In this sense, TEM analysis was chosen to explore the size and morphology of our AgNPs. TEM image (inset of Figure 1b) and size distribution analysis (Figure 1b) clearly show non-agglomerated spherical nanoparticles with a predominant diameter between 10 and 15 nm. This result proves the efficiency of PVA as a capping agent to prevent nanoparticle self-aggregation. Actually, it was demonstrated that PVA interacts with the silver particle surface by physical adsorption through the oxygen atoms, avoiding their agglomeration [36].

The effective interaction of PVA with silver nanoparticles was also studied by Fourier transform infrared spectroscopy (FTIR) (Figure 1c). FTIR spectra of pure PVA powder and silver nanoparticles both without (“nude particles”, NAgNPs) and with PVA (AgNPs) were performed. When comparing the spectra of NAgNPs and AgNPs, it is evident the changes in the intensity of the vibrational bands at 2930, 1720, 1423, 1323, 1070, and 875 cm^−1^ that correspond to CH_2_ asymmetric stretching vibration, C=O stretch, C-H bending vibration of CH_2_, C–H deformation vibration, C–O stretching of acetyl groups, and C–C stretching vibration of PVA, respectively [37], suggesting that the polymer is certainly adsorbed onto the surface of the metallic particles.

#### Antimicrobial Activity of Silver Nanoparticles

**Figure 2:**
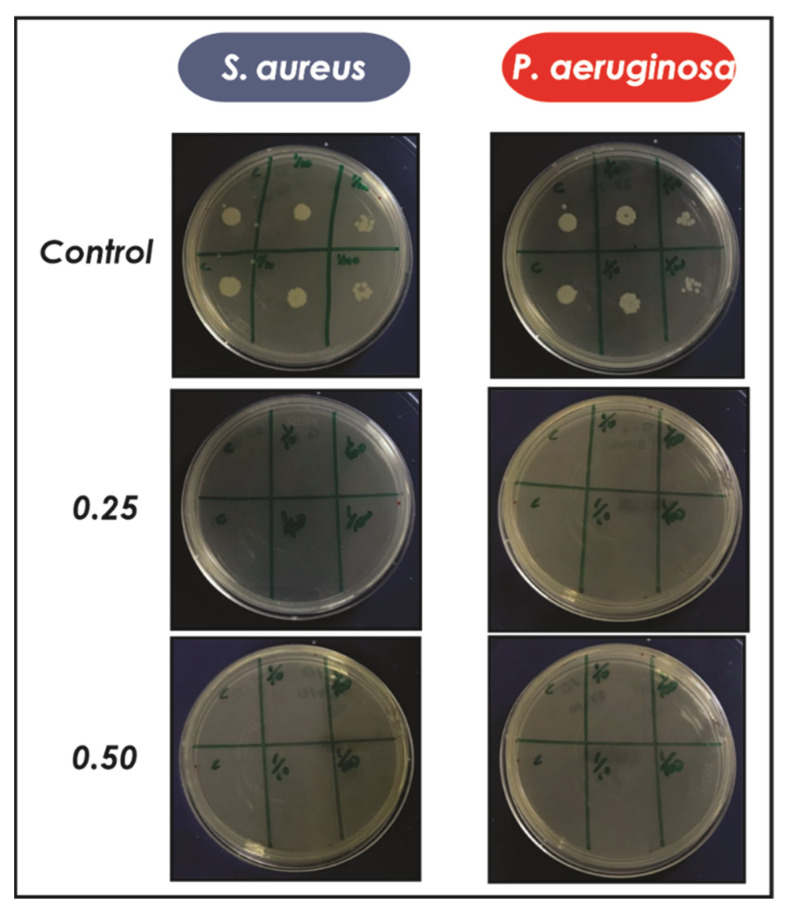
Antimicrobial activity of AgNPs against *S. aureus* and *P. aeruginosa* for different ratios of AgNPs/inoculum (0.25 and 0.50). After 24 h at 37 °C, three different dilutions were seeded in Petri dishes in duplicate. Finally, the viability of the microorganisms was evaluated at 24 h.

With the aim of developing an antimicrobial material, the bactericidal activity of AgNPs was evaluated against Gram-negative (*P. aeruginosa*) and Gram-positive (*S. aureus*) bacteria. For this, a known concentration of silver nanoparticles was added to the culture of each bacterium with a ratio of AgNPs/inoculum 0.25 and 0.50 and after 24 h different aliquots were extracted and seeded in Petri dishes. Finally, the growth of the bacteria was studied after 24 h. Figure 2 clearly shows a complete inhibition of the growth of *P. aeruginosa* and *S. aureus* when they were treated with AgNPs, compared to the control without nanoparticles that exhibited an important bacterial proliferation at all dilutions evaluated.

### 2.2. Preparation and Characterization of Antimicrobial Hydrogels

**Figure 3:**
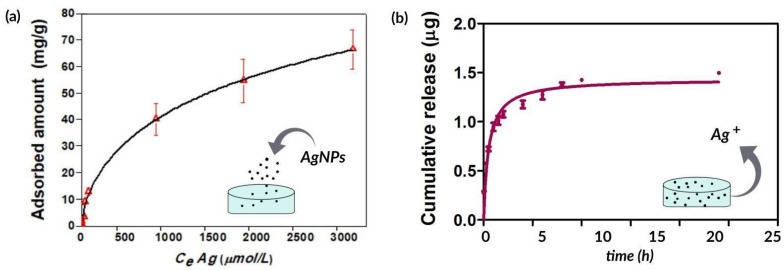
(**a**) Adsorption isotherm of AgNPs on collagen matrices. *CeAg (μmol/L)* corresponds to the equilibrium concentration of Ag. (**b**) Cumulative release of Ag^+^ from collagen over 24 h. Results are expressed as mean ± SD from triplicate experiments.

Once AgNPs were prepared and characterized, they were incorporated in collagen hydrogels in order to develop an antimicrobial scaffold for wound healing. Unlike other methods that use chemical crosslinking agents to induce gelation of collagen, in this work, the hydrogels were obtained by incubating an acidic collagen solution in saturated ammonia (NH_3_) chamber for 12 h, and subsequently washing several times with water until neutral pH is reached [6]. This ensures the conservation of the biocompatibility of the gels and avoids the possible toxic effects of common chemical crosslinkers [38]. For loading silver nanoparticles into collagen hydrogels, different known concentrations of AgNPs were added to the biomaterial and the adsorption isotherm was evaluated after 48 h. Subsequently, as these assemblies might be used to prevent infections in skin wounds, the release kinetics of silver ions (Ag^+^) from AgNPs incorporated into the collagen hydrogels was studied. According to Figure 3, 67 ± 7 mg of AgNPs is adsorbed per g of collagen (Figure 3a) and only 1.5 μg of the silver content is released after 24 h (Figure 3b). This result is worth noting as a controlled release of the bactericidal silver ions from collagen hydrogels is critical to achieving a long-lasting antimicrobial material.

**Figure 4:**
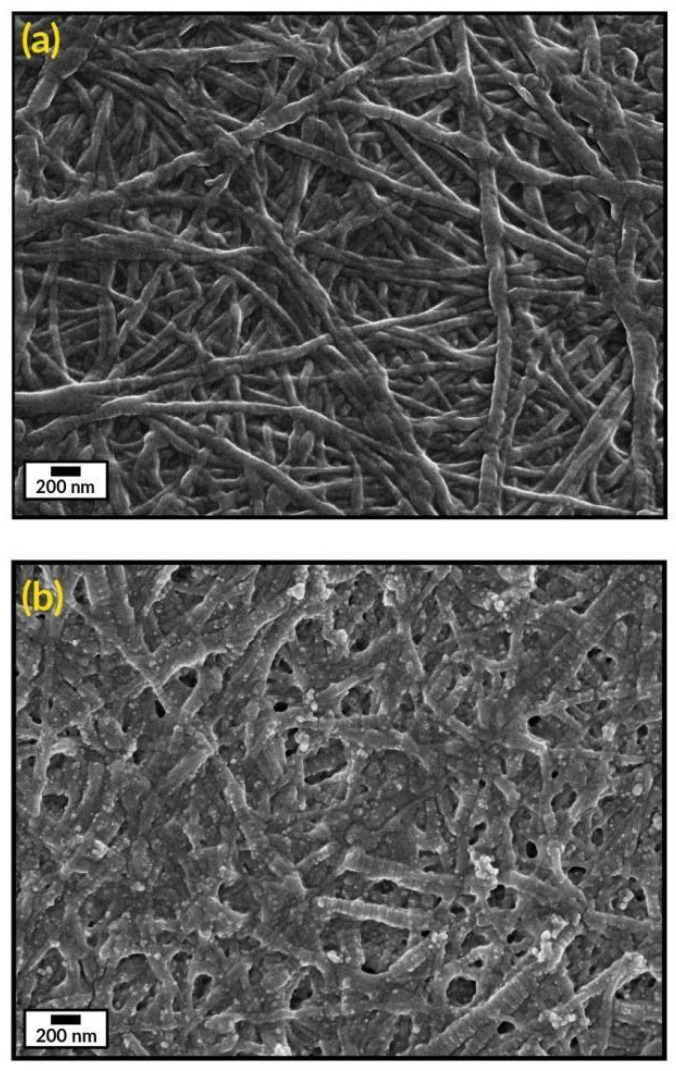
SEM images of (**a**) collagen hydrogel (Col) and (**b**) collagen gel containing silver nanoparticles (Col-AgNPs). Scale bars represent 200 nm.

The microstructure of the collagen gels (Col) and the biocomposites (Col-AgNPs) was analyzed by Scanning electron microscopy (SEM). Figure 4a illustrates the representative morphology of collagen hydrogels (8 mg/mL protein concentration) composed of densely packed fibrils/fibers, which are characterized by a pattern of regular bands with a D-period of 65 nm, typical of collagen type I of vertebrates [39]. It was reported that this particular structure of fibrillar collagen arises from a right-handed bunch of three parallel, left-handed polyproline helices of the collagen molecules [40]. This characteristic morphology of collagen was not modified after the incorporation of silver nanoparticles. Figure 4b reveals that AgNPs keep attached to collagen fibrils and spread widely throughout the material after they are adsorbed onto the gels. This can be explained by the formation of hydrogen bonds between the biopolymer and OH groups of the capping agent (PVA) that surrounds the nanoparticles [41].

**Figure 5:**
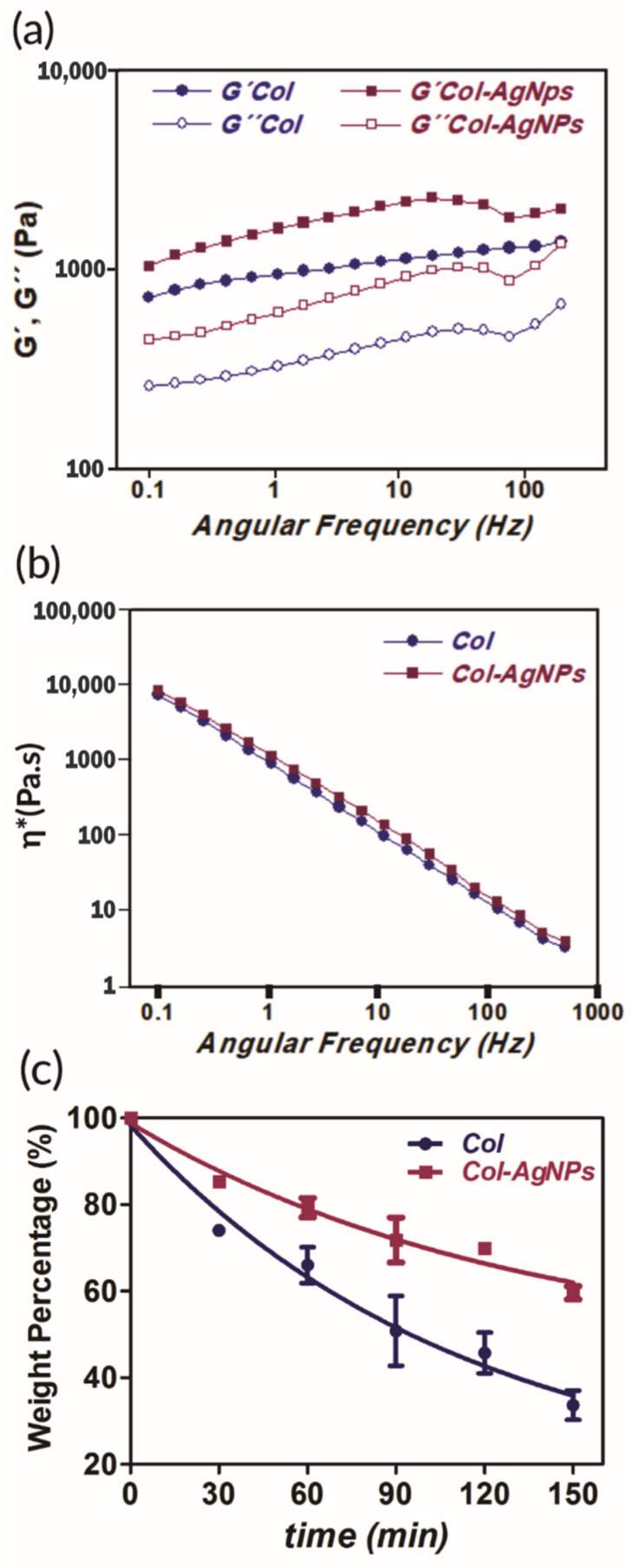
Mechanical properties (**a**), (**b**) and stability (**c**) of collagen scaffolds. (**a**) Dependence of the elastic (G′) and viscous modulus (G″) with frequency and (**b**) the viscosity (η*) of Col-AgNPs 67 mg/g (pink) and Col control (blue) at 37 °C. (**c**) Collagenase degradation of collagen matrices. Weight percentage is shown as a function of time for Col control (blue) and Col-AgNPs 67 mg/g (pink). Results are expressed as mean ± SD from triplicate experiments.

Mechanical properties and stability of collagen composites were also examined. The study of these features of a new biomaterial under development is a fundamental step for the success of clinical applications. For this reason, we studied the rheological behavior of Col and Col-AgNPs at body temperature (37 °C) with the aim of verifying the stiffness of the hydrogel with or without the addition of silver nanoparticles. According to the results of Figure 5a, both materials possess a gel-like behavior since G′ elastic components presented higher values than G″ viscous ones. Furthermore, Col-AgNPs are twice as elastic as unmodified Col hydrogel, indicating that AgNPs play a crucial role as a reinforcing component. This can be justified by a dual effect of the AgNPs: on one hand, they act as inorganic fillers and, at the same time they interact by hydrogen bonds with the collagen scaffold, resulting in enhanced mechanical properties. Besides, Figure 5b shows that the viscosity of Col and Col-AgNPs decays with increasing angular frequency revealing non-Newtonian behavior characteristics of pseudoplastics [42]. This is imperative for the design and construction of new biomaterials that need to be compatible with the tissue at the site of application. Indeed, many studies that developed different methods to improve mechanical properties of gels made from low collagen concentration have been reported, resulting in stiff and stable hydrogels [43,44].

In this context, the stability of Col and Col-AgNPs was also analyzed by a metalloproteinase degradation assay. For this, the collagen scaffolds were incubated with the enzyme collagenase (20 U/mL) at 37 °C for 2.5 h. While pure collagen (Col) conserved only 44% of its original weight, the hydrogel containing AgNPs preserved around 60% of its initial mass (Figure 5c). Taking this into account and considering rheology results, it is clear that AgNPs enhance the mechanics and improve the stability of the hydrogels through strong interactions between PVA and amino groups of collagen fibers, inhibiting collagenase degradation. This reinforcement of the mechanical parameters of the Col-AgNPs biocomposite is really beneficial to prolong the utility of the dressings in contact with the wounds.

#### 2.2.1. Antimicrobial Activity of Col-AgNPs

**Figure 6:**
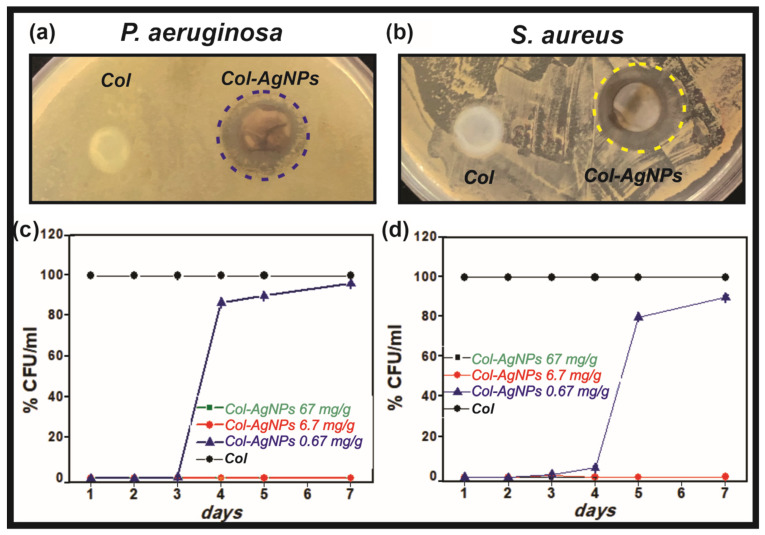
Antimicrobial activity Col-AgNPs against *S. aureus* and *P. aeruginosa*. Collagen hydrogels containing AgNPs in contact with *P. aeruginosa* (**a**) and *S. aureus* (**b**). The % CFU/mL of each bacterium was studied during 7 days (**c**,**d**) for collagen control (Col, black), Col-AgNPs 67 mg/g (green), Col-AgNPs 6.7 mg/g (red) and Col-AgNPs 0.67 mg/g (blue). Results are expressed as mean ± SD from triplicate experiments.

Considering the controlled release of bactericidal Ag^+^ ions from Col-AgNPs, antimicrobial activity of the hybrid collagen gels was studied against Gram-positive (*S. aureus*) and Gram-negative (*P. aeruginosa*) bacteria. For this, the disk diffusion test was used as a preliminary test to evaluate the bactericidal activity of Col-AgNPs at 24 h. For antibiograms, a bacterial suspension was spread on an agar plate and the collagen scaffolds with and without AgNPs were placed on the agar surface and incubated for 24 h at 37 °C. Finally, the diameter of the inhibition zones around the gels was measured after incubation. Figure 6a,b clearly show the inhibitory effect of Col-AgNPs on the growth of both bacteria, delimiting well-defined bacteria-free zones around the hybrid hydrogels. However, the control collagen gels had no inhibitory effect on bacteria colonization.

The antimicrobial activity of Col-AgNPs was also studied by the dilution method in order to evaluate the bactericidal effect of the material over time (from 24 h to 7 days). This method was also used in a previous work [7] in which silver nanoparticles were first incorporated in liposomes and then in collagen scaffolds (Col-L-AgNPs). Briefly, the collagen hydrogels containing three different concentrations of AgNPs (0.67, 6.7, and 67 mg/g) were incubated with a 1 × 10^5^ CFU/mL bacterial suspension for 24 h and up to 7 days at 37 °C. After each time, the number of colonies of the corresponding supernatants was determined on agar Petri plates and CFU/mL was finally calculated. Figure 6c,d shows that Col-AgNPs 67 and 6.7 mg/g cause a major inhibition of the growth of *P. aeruginosa* (Gram-negative) and *S. aureus* (Gram-positive) for seven days, since no colony was developed. However, when the concentration of AgNPs was reduced to 0.67 mg/g, the bactericidal activity was effective only until the third day and then an increase in the number of bacterial colonies was observed. This result is especially interesting as it demonstrates that the bacterial colonization of our hybrid collagen scaffold can be regulated over time with the concentration of the antimicrobial agent. A similar behavior was observed for the Col-L-AgNPs bactericidal effect which depends on the concentration of liposomal AgNPs added to collagen hydrogels, but also on the bacteria strain [7].

#### 2.2.2. In Vitro Cytotoxicity of Col-AgNPs

Biocompatibility is a crucial factor to consider when designing a new material to promote wound healing without any negative response from the host if future clinical application is desired. For this reason, cytotoxicity of Col-AgNPs towards Madin-Darby Canine Kidney (MDCK) epithelial cells was studied by the colorimetric 3-(4,5-dimethylthiazol-2-yl)-2,5-diphenyl-2H-tetrazolium bromide (MTT) assay. Taking into consideration the antimicrobial activity obtained for collagen composites, cell viability of Col-AgNPs 0.67, 6.7, and 67 mg/g was evaluated during 72 h of treatment, the period in which they exhibited significant bactericidal effect.

**Figure 7:**
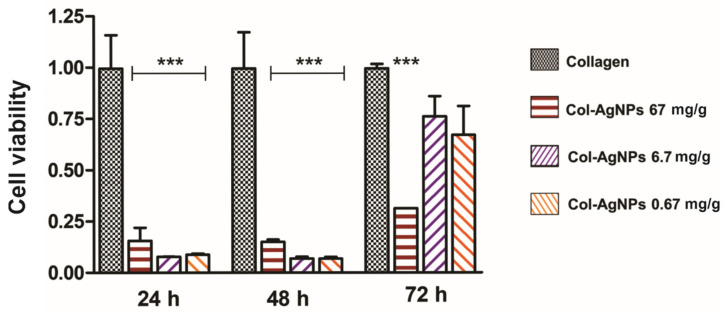
Viability of MDCK epithelial cells evaluated by the MTT test. Cell viability is plotted as a function of time for Col (black), Col-AgNPs 67 mg/g (red), Col-AgNPs 6.7 mg/g (violet) and Col-AgNPs 0.67 mg/g (orange). Viability rate in pure collagen hydrogels were considered as 1 at each time tested. Results are expressed as mean ± SD from triplicate experiments. *** Statistical significance (*p* < 0.001).

In Figure 7, the cell proliferation rate as a function of time is plotted for each of the evaluated materials. It can be observed that cell proliferation was detected in all the materials during the three days were tested. However, compared to Col control, cell viability obtained for Col-AgNPs 0.67, 6.7, and 67 mg/g was significantly lower during the first 48 h. On the third day, cell proliferation remained inhibited for Col-AgNps 67 mg/g, while for Col-AgNPs 0.67 and 6.7 mg/g it showed an important increase. This is indicating that Col-AgNPs inhibit cell growth probably due to the amount of accumulated silver ions that may be toxic for the living epithelial cells [45]. Consequently, it was imperative to add some other components to enhance cell proliferation and improve the biocompatibility of the antimicrobial collagen composites.

### 2.3. Preparation and Characterization of Antimicrobial Hydrogels with Cannabis Sativa Oil

#### 2.3.1. Antioxidant Activity

In order to reduce cytotoxicity of the antimicrobial scaffolds based on collagen and silver nanoparticles, a *Cannabis sativa* oil was added to the hydrogels. It was demonstrated that AgNPs stimulate the production of reactive O_2_ species inducing oxidative stress in cells and thus inhibiting their proliferation [46]. In this sense, as *Cannabis sativa* has shown remarkable antioxidant capacity [47] stimulating wound healing, we added this natural extract to Col-AgNPs. Firstly, we studied the antioxidant activity of two different amounts of *Cannabis sativa* extracted from White widow plants before its incorporation into the hydrogels. Table 1 shows that a higher amount of the oil has a strong scavenger activity of the 2,2-diphenyl-1-picrylhy-drazyl (DPPH) radical. Consequently, this 150 µL volume of *Cannabis sativa* extract was selected to be added to the collagen and silver nanoparticles gels.

It was crucial now to study the antioxidant activity of the new hydrogels containing *Cannabis sativa* (CS) oil (Col-CS-AgNPs). Table 2 shows that Col-AgNPs have a low capacity of inhibiting DPPH radical, but after incorporation of *Cannabis sativa* oil, this antioxidant activity percentage increased significantly. This result demonstrates that CS conserves its antioxidant properties after its inclusion in the hydrogels and that it is not affected by the presence of AgNPs. This behavior is essential to improve the biocompatibility of the material.

#### 2.3.2. Biocompatibility Col-AgNPs with Cannabis Sativa Oil

**Figure 8:**
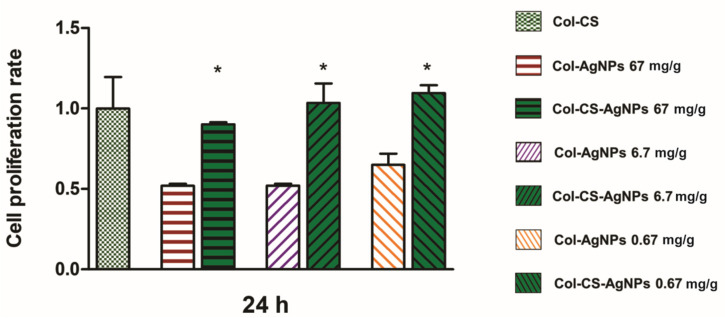
Viability of MDCK epithelial cells evaluated by the MTT test. Cell proliferation rate is plotted as a function of time for Col-CS (light green), Col-AgNPs 67 mg/g (red), Col-AgNPs 6.7 mg/g (violet), Col-AgNPs 0.67 mg/g (orange), Col-CS-AgNPs 67 mg/g (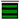), Col-CS-AgNPs 6.7 mg/g (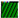), Col-CS-AgNPs 0.67 mg/g (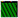). Viability rate in Col-CS were considered as 1. Results are expressed as mean ± SD from triplicate experiments. * Statistical significance (*p* < 0.05).

Once the antioxidant activity of Col-CS-AgNPs was confirmed, we studied the biocompatibility of the bactericidal collagen scaffolds. For this, 150 µL of *Cannabis sativa* oil was added to previously synthesized Col-AgNPs (67, 6.7, 0.67 mg/g) and cytotoxicity of these new materials (Col-CS-AgNPs) towards MDCK epithelial cells was evaluated for 24 h. Figure 8 shows a significant increase in cell proliferation rate for materials containing *Cannabis sativa* compared to collagen hydrogels that only include silver nanoparticles. This can be explained by the antioxidant properties of the *Cannabis sativa*. As it reduces the concentration of ROS produced by cells in contact with AgNPs, oxidative stress decreases and cell proliferation is stimulated. This result is really promising since it offers the opportunity to develop a new biomaterial with high antimicrobial activity while maintaining its biocompatibility.

#### 2.3.3. Antimicrobial Activity Col-CS-AgNPs

**Figure 9:**
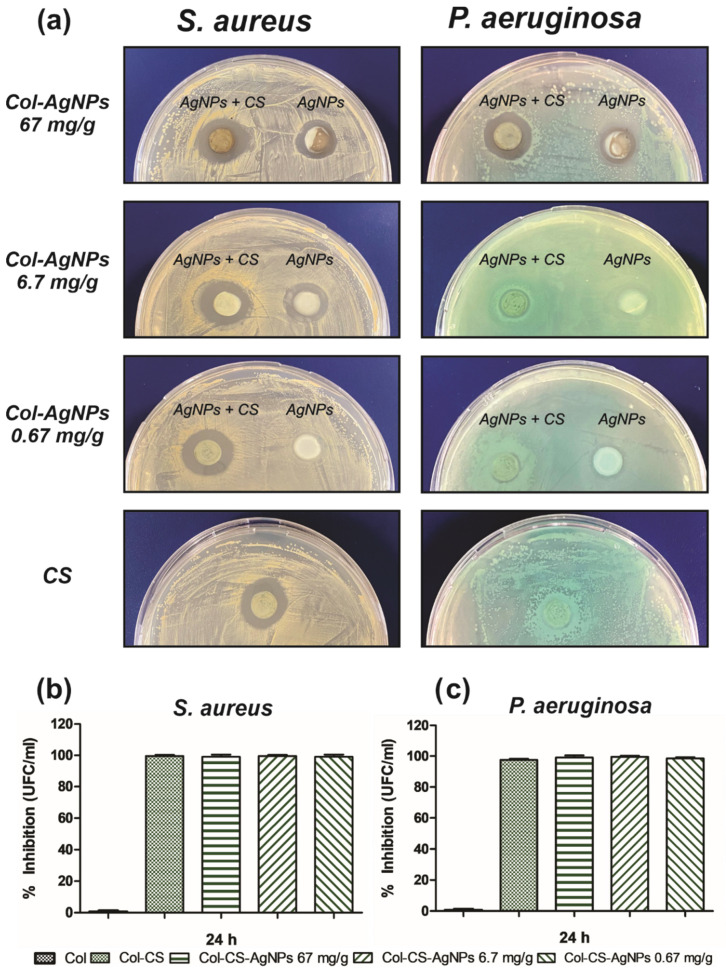
Antimicrobial activity of Col-CS-AgNPs against *S.aureus* and *P. aeruginosa*. (**a**) Disk diffusion method for Col-CS and Col-AgNPs 67, 6.7 and 0.67 mg/g containg or not CS, against *S. aureus* and *P. aeruginosa*. (**b**) Percentage of inhibition of the growth of *S. aureus* (**b**) and *P. aeruginosa* (**c**) evaluated by the dilution method.

Since the early findings, plenty of reports on the antimicrobial activity of cannabinoids have been published [19]. In most of them, cannabis extracts showed potent bactericidal effects against primarily Gram-positive bacteria, but minor activity against Gram-negative bacteria. In this sense, we study the effect of the incorporation of CS to Col-AgNPs on the antimicrobial activity of the hydrogel against *S. aureus* and *P. aeruginosa*. For this, the disk diffusion test was firstly performed using the same methodology as previously described. After 24 h, the diameter of the inhibition zones around the gels was measured. Figure 9a confirms the antimicrobial activity of CS against *S. aureus* giving an inhibition zone of 1.58 ± 0.08 cm. Furthermore, CS enhances the bactericidal effect of Col-AgNPs 67, 6.7 and 0.67 mg/g as the diameter of the inhibition zones increased from 1.45 ± 0.07, 1.35 ± 0.07 and 1.5 cm to 1.75 ± 0.06, 1.85 ± 0.07 and 1.65 ± 0.05 cm, respectively, after adding the *Cannabis sativa* oil extract to the hydrogels. This suggests an antimicrobial synergistic effect of AgNPs and CS against *S. aureus*. On the other hand, as it was expected, Col-CS has no inhibition effect on the growth of *P. aeruginosa*. However, hydrogels containing AgNPs and CS did show antimicrobial activity against this Gram-negative bacteria, indicating that *Cannabis sativa* oil does not improve the antimicrobial capacity of AgNPs. This distinct antimicrobial power observed over Gram-positive and Gram-negative bacteria may be explained by the mechanism of action proposed for cannabidiols [21]. It has been suggested that cannabidiols inhibit the synthesis of proteins, DNA, RNA and peptidoglycan and rapidly disrupt cytoplasmic membranes in Gram-positive but less in Gram-negative bacteria.

When the antimicrobial activity of Col-CS-AgNPs was tested by dilution method, the percentage of inhibition of the growth of bacteria was higher than 99% in all cases, both for Gram-positive (Figure 9b) and Gram-negative (Figure 9c) bacteria. Interestingly, Col-CS now presented an inhibition of 98.37% for *P. aeruginosa*. This result that indicates a more effective action of antimicrobial agents in the liquid medium than in the solid medium of the antibiograms, evidences the bactericidal potential of Col-CS-AgNPs and Col-CS against a broad spectrum of bacteria. Besides, the synergistic effect observed from the combination of AgNPs with CS could improve the antimicrobial properties of our material over time.

## 3. Materials and Methods

### 3.1. Preparation of Silver Nanoparticles

In order to produce the silver nanoparticles (AgNPs), silver nitrate was reduced with sodium borohydride (NaBH_4_) in the presence of PVA, a capping agent, to prevent particles agglomeration. Briefly, 10% PVA was mixed with 80 mM NaBH_4_ in an ice bath. A solution of 40 mM AgNO_3_ was then added drop by drop under constant stirring. After all the AgNO_3_ had been added, the obtained colloidal suspension was centrifuged in order to remove the larger particles that could have been formed. The pellet was discarded, and the supernatant was stored at 4 °C [48]. The electrothermal atomization method using pyrolytic graphite tubes was used to determine the final silver concentration using Atomic Absorption Spectrometry in a 210 VGP spectrometer (Buck Scientific, Norwalk, CT, USA).

### 3.2. Preparation of Collagen Gels

Type I collagen was obtained from rat tail tendons and stored in acetic acid solution at 4 °C. Using the Bergman method, hydroxyproline titration was used to estimate the collagen concentration [49]. In order to prepare the collagen gels, 0.2 mL of the acid collagen solution was added into 48 well flat-bottom plates, and to avoid collagen denaturation, the temperature was maintained below 10 °C. To induce gelation plates were placed in an ammonia vapor chamber at room temperature. After 12 h, the obtained gels were removed and, under sterile conditions, the excess of ammonia was evaporated. Lastly, in order to reach a neutral pH, collagen gels were washed three times with distilled water.

### 3.3. Preparation of the Scaffolds with AgNPs

In order to prepare the collagen-based scaffolds, 0.150 mL of different concentrations of AgNPs were added to the collagen gels (Col-AgNPs). The excess was removed after 48 h and the gels were transferred to a clean 24-well plate.

### 3.4. Preparation of the Hybrid Scaffolds with Cannabis Sativa Oil Extract

In order to prepare the hybrid scaffolds (Col-CS), 0.150 mL of *Cannabis sativa* oil extract (2.4957 mg/mL cannabidiol (CBD), 10.0910 mg/mL ∆9-tetrahydrocannabinol (THC) and 0.7561 mg/mL cannabigerol (CBG)) was added to the gels previously described in Section 2.3 (Col-CS-AgNPs).

### 3.5. Characterization

#### 3.5.1. Physical Characterization

In order to assess the optical properties of AgNPs, a suspension of silver nanoparticles was monitored by UV–vis spectroscopy using a Beckman DU 7400-diode array spectrophotometer. In order to study the morphology of AgNPs, transmission electron microscopy (TEM) was performed using a Zeiss EM109T electron microscope. Briefly, a drop of the sample was added to carbon-copper grids and allowed to dry for a few minutes. To analyze the collagen morphology, scanning electron microscopy (SEM) was performed using a Carl Zeiss NTS-Supra 40 microscope. In this sense, a 2.5% glutaraldehyde solution was used to fix the gels for 1 h at 4 °C and after that, gels were washed three times with distilled water. Finally, samples were freeze-dried and gold sputter-coated prior to analysis. Fourier transform infrared (FTIR) spectra of AgNPs and pure PVA was performed using 0.150 mL of the sample over the range of 4000–500 cm^−1^, using an FTIR-Raman Nicolet iS 50 (Thermo Scientific, Waltham, MA, USA). Briefly, the samples were dried under a nitrogen flow, then the powder was placed on the attenuated total reflection accessory of the spectrometer and the spectra were recorded.

#### 3.5.2. Silver Adsorption and Release Studies

Hydrogel’s equilibrium adsorption capacity was evaluated by adding 0.15 mL of different concentrations of AgNPs solutions to the collagen gels. The samples were equilibrated at room temperature for 48 h, the non-retained adsorbates were removed, and atomic absorption spectroscopy was used to measure their silver content. In order to calculate the equilibrium sorption capacity, the following equation was used:

(1) $$qe=\left(\frac{C_{0}-C_{e}}{m}\right)V$$

where *qe* is the amount of sorbed AgNPs per g of collagen, *C*_0_ and *C_e_* are the initial and the equilibrium concentrations, respectively, *m* is the collagen weight (1.6 mg) and *V* is the total volume (0.5 mL). Silver ions (Ag^+^) released from AgNPs were evaluated using 5 mL of suspension. Briefly, the sample was placed in the upper chamber of Vivaspin^®^ 20 centrifugal concentrator (30 kDa molecular weight cutoff, Sartorius Stedim Biotech GmbH, Göttingen, Germany) and was centrifuged at 5000 rpm for 15 s at 25 °C. After this, the aqueous filtrate contained the released Ag^+^, whereas the nanoparticles remained in the upper chamber. Atomic absorption was used to measure the Ag concentration in the filtrate for 24 consecutive hours. In order to quantify the release of Ag^+^ from the AgNPs contained in the collagen gels, hydrogels were soaked in 0.5 mL of deionized water. After that, atomic absorption was used to measure the Ag concentration in the gel supernatant for 24 consecutive hours. A calibration curve was performed in order to calculate the cumulative doses over time. Results were expressed as mean ± SD from triplicate experiments.

### 3.6. Rheological Properties

In order to study the rheological behavior of the materials, first amplitude sweeps were performed to determine the linear viscoelastic range. Using a rotational rheometer from Anton Paar (MCR-301) provided with a CTD 600 thermo chamber the following parameters of the studied materials were obtained in small-amplitude oscillatory shear flow experiments: the elastic or storage modulus, G′ (ω), the viscous or loss modulus, G″ (ω) and complex viscosity (η*). The study was performed at 37 °C using parallel plates of 25 mm diameter and 0.1–500 s^−1^ range of frequency. To ensure the dynamic response linearity, small strains (γ = 1%) were used in all the test. The gap width used was 800–1000 μm.

### 3.7. Enzymatic Degradation of Collagen Gels

The degradation of the collagen gels with AgNPs by collagenase digestion was evaluated. Briefly, the collagen gels were incubated with a collagenase enzyme solution (1 mL of a 20 U/mL solution of type I collagenase Gibco^®^, 260 U/mg) at 37 °C. The weight of the collagen gels was measured at different times.

### 3.8. Total Antioxidant Capacity

DPPH colorimetric assay was used to study the antioxidant activity of the *Cannabis sativa* oil extract and Col-CS-AgNPs [50]. The method consisted in the scavenging activity of the stable 2,2-diphenyl-1-picrylhydrazyl free radical (DPPH•). For *Cannabis sativa*, 10 μL and 150 μL of the oil extract was incubated with 3 mL of the methanolic solution of DPPH• (25 mg/L). In the case of Col-CS-AgNPs, the hydrogels were incubated with 3 mL of the methanolic solution of DPPH• (25 mg/L) for 5 min. In both cases, the absorbance was measured at 517 nm. The percentage of inhibition was calculated by the Equation:

%inhibition = [1 − (Abssample/AbsDPPH solution)] × 100(2)

### 3.9. Antimicrobial Activity

Bactericidal activity of AgNPs, Col-AgNPs, Col-CS and Col-CS-AgNPs was evaluated on Gram-negative (*P. aeruginosa*) and Gram-positive (*S. aureus*) bacteria. *S. aureus* (ATCC 29213) and *P. aeruginosa* (ATCC 27853) were grown overnight at 37 °C in Luria Bertani (LB) medium (yeast extract, 5 g/L; NaCl, 10 g/L and tryptone, 10 g/L) and diluted obtaining a concentration of 1 × 10^6^ Colony Forming Units (CFU)/mL. Different concentrations of AgNPs were incubated with a certain amount of *S. aureus* and *P. aeruginosa* in LB medium at 37 °C for 24 h. After this time, 20 μL of the samples were plated on agar Petri dishes and after 24 h the bacterial growth was studied.

*S. aureus* and *P. aeruginosa* were also used to evaluate the antimicrobial activity of Col-AgNPs, Col-CS, and Col-CS-AgNPs by two different methods, disk diffusion method for antibiograms and the dilution method. For the first method, bacterial suspension, from a 1:1000 culture dilution, was spread on an agar Petri dish. After 10 min of incubation at room temperature, Col-AgNPs, Col-CS, and Col-CS-AgNPs were placed on the agar surface. The inhibitory effect of the gels was evaluated after 24 h by measuring the diameter of the bacteria-free zones surrounding the gels.

In order to evaluate the antimicrobial activity using the dilution method, 0.1 mL of the bacterial suspension, from the 1:1000 culture dilution, were added to Col-AgNPs, Col-CS, and Col-CS-AgNPs with 0.2 mL of PBS solution. The gels were incubated at 37 °C from 24 h up to 7 days. In this sense, the supernatants were withdrawn and serial dilutions were performed in PBS solution. 20 μL aliquots of the dilutions were plated on agar Petri dishes and incubated overnight at 37 °C. In order to determine the number of CFU, colonies on the plates were manually counted. Results were expressed as mean ± SD from triplicate experiments.

### 3.10. Cytotoxicity Experiments

Biocompatibility was studied using MDCK epithelial cell line (Madin-Darby Canine Kidney). Briefly, this cell line was grown in adherent culture flasks with Dulbecco’s Modified Eagle’s Medium, supplemented with 10% fetal calf serum, 0.25 μg/mL fungizone and 1% penicillin–streptomycin and maintained at 37 °C in a humidified chamber (95% air; 5% CO_2_) until confluence was reached. In order to perform the experiments, trypsin–EDTA solution was used to detach the cells. To assess cellular viability 1 × 10^4^ cells (previously counted using a Neubauer camera and trypan blue staining) were added on top of each gel with 0.5 mL of cell culture medium and were maintained at 37 °C in a humidified chamber (95% air; 5% CO_2_). The biocompatibility of collagen gels with AgNPs was studied using the colorimetric 3-(4,5-dimethyl-thiazol-2-yl)-2,5-diphenyl-tetrazolium bromide (MTT) assay at 24 h, 48 h, and 72 h. Hybrid scaffolds with *Cannabis sativa* oil extract biocompatibility was studied at 24 h. Briefly, the medium was removed and a 5 mg/mL MTT solution was added. After the incubation for 4 h at 37 °C, the MTT solution was removed, the gels were washed three times with PBS and absolute ethanol was added. The measure was performed at 570 nm and results were expressed as mean ± SD from triplicate experiments.

### 3.11. Statistical Analysis

Data are expressed as means ± SD of triplicate experiments. The differences were analyzed using one-way ANOVA, followed by the Tuckey’s post-test; *p* < 0.05 was considered significant.

## 4. Conclusions

In this work, a novel biomaterial with high and long-term antimicrobial effects against Gram-positive (*S. aureus*) and Gram-negative (*P. aeruginosa*) bacteria was developed. The incorporation of silver nanoparticles in collagen hydrogels synthesized without the use of chemical crosslinking agents resulted in a sustained release of silver ions, leading to prolonged antimicrobial activity. Moreover, our bactericidal material exhibited enhanced mechanical properties due to the addition of AgNPs and showed increased stability against enzymatic collagenase degradation which could encourage the application of the dressings.

On the other hand, the combination of this antimicrobial collagen hydrogel with *Cannabis sativa* oil allowed to improve the biocompatibility of the scaffold which is crucial for its dermal use for wound healing. Furthermore, as *Cannabis sativa* is known for its anti-inflammatory, analgesic, and antimicrobial effects, its incorporation in our bactericidal collagen gel is interesting to think about a future clinical application for the treatment of skin wounds.

In conclusion, since collagen has been widely applied for tissue regeneration, this new multifunctional biomaterial is an attractive option for preventing injuries infections and favoring wound repairing.

## Figures and Tables

**Table 1:** Antioxidant activity of *Cannabis sativa* oil extract calculated as the inhibition percentage of DPPH radical.

Sample	Scavenger Activity (%)
*Cannabis sativa* oil extract (10 µL)	16.00 ± 1.01
*Cannabis sativa* oil extract (150 µL)	80.00 ± 1.52

**Table 2:** Antioxidant activity of collagen hydrogels loaded with silver nanoparticles and *Cannabis sativa* oil extract calculated as the inhibition percentage of DPPH radical.

Sample	Scavenger Activity (%)
Col	4.20 ± 1.14
Col-AgNPs 67 mg/g	7.57 ± 1.52
Col-AgNPs 6.7 mg/g	6.12 ± 1.16
Col-AgNPs 0.67 mg/g	7.94 ± 1.19
Col-CS	47.20 ± 1.15
Col-CS-AgNPs 67 mg/g	42.23 ± 1.18
Col-CS-AgNPs 6.7 mg/g	42.06 ± 1.20
Col-CS-AgNPs 0.67 mg/g	41.07 ± 1.19

## Data Availability

Data is contained within the article.
